# Iron Chelators in Treatment of Iron Overload

**DOI:** 10.1155/2022/4911205

**Published:** 2022-05-05

**Authors:** Sarina Entezari, Seyedeh Mona Haghi, Narges Norouzkhani, Barsa Sahebnazar, Fatemeh Vosoughian, Diba Akbarzadeh, Muhammad Islampanah, Navid Naghsh, Mohammad Abbasalizadeh, Niloofar Deravi

**Affiliations:** ^1^Student Research Committee, School of Allied Medical Sciences, Shahid Beheshti University of Medical Sciences, Tehran, Iran; ^2^Student Research Committee, School of Pharmacy, Mashhad University of Medical Science, Mashhad, Iran; ^3^Department of Medical Informatics, Faculty of Medicine, Mashhad University of Medical Sciences, Mashhad, Iran; ^4^Student Research Committee, Fasa University of Medical Sciences, Fasa, Iran; ^5^Student Research Committee, School of Medicine, Shahid Beheshti University of Medical Sciences, Tehran, Iran; ^6^Faculty of Medicine, Mashhad University of Medical Sciences, Mashhad, Iran; ^7^Department of Pharmacy, Shahid Sadoughi University of Medical Sciences, Yazd, Iran; ^8^Faculty of Medicine, Tabriz University of Medical Sciences, Tabriz, Iran

## Abstract

Patients suffering from iron overload can experience serious complications. In such patients, various organs, such as endocrine glands and liver, can be damaged. Although iron is a crucial element for life, iron overload can be potentially toxic for human cells due to its role in generating free radicals. In the past few decades, there has been a major improvement in the survival of patients who suffer from iron overload due to the application of iron chelation therapy in clinical practice. In clinical use, deferoxamine, deferiprone, and deferasirox are the three United States Food and Drug Administration-approved iron chelators. Each of these iron chelators is well known for the treatment of iron overload in various clinical conditions. Based on several up-to-date studies, this study explained iron overload and its clinical symptoms, introduced each of the above-mentioned iron chelators, and evaluated their advantages and disadvantages with an emphasis on combination therapy, which in recent studies seems a promising approach. In numerous clinical conditions, due to the lack of accurate indicators, choosing a standard approach for iron chelation therapy can be difficult; therefore, further studies on the issue are still required. This study aimed to introduce each of these iron chelators, combination therapy, usage doses, specific clinical applications, and their advantages, toxicity, and side effects.

## 1. Introduction

Iron is a vital component of the human body, and its concentration is strictly regulated. While iron is essential for life, it can also be toxic to the cells [1]. Iron overload is associated with multiorgan damage [2]. This is due to a lack of ways to protect human cells from iron overload and iron function in the production of free radicals [3, 4]. Iron overload occurs when iron intake is increased over time, whether as a result of red blood cell transfusions, increased iron absorption through the gastrointestinal tract, repeated transfusions, iron misuse often as a supplement, chronic hepatitis, or hereditary hemochromatosis [5–9].

The transfusions of red blood cells are becoming more common in the treatment of different anemias. Transfusion iron overload has a strong relationship with the number of blood transfusions. Each unit of transfused blood consists of 200–250 milligrams of iron, and since human beings are not physiologically capable of excreting extra iron, blood transfusion therapy leads to the accumulation of iron in the body. Therefore, iron overload is a substantial concern to patients receiving about 10 to 20 units of blood and even more. Patients becoming transfusion dependent with different types of anemia, such as aplastic anemia, thalassemia, sickle cell disease (SCD), hemolytic anemia, and myelodysplastic anemia, undoubtedly develop iron overload in the future, and this can cause harmful effects to the body [10, 11].

Iron toxicity leads to free radical production, which causes severe side effects [3], such as cardiac dysfunctions, including arrhythmia and cardiomyopathy, hemosiderosis [12], delays of sexual maturity, liver cirrhosis, liver cancer, hepatitis [2], impotence, infertility [13], and metabolism dysfunction [14]. It is important to suppress labile plasma iron and eliminate excess iron to prevent its severe complications [15]. Iron-chelating agents have been introduced in medical care to protect patients from the effects of iron toxicity [16]. They bind metal ions and reduce their reactivity dramatically [17]. Therefore, iron-chelating agents reduce iron overload in different organs, such as the liver and heart, and prevent complications, such as endocrinological, hepatic, and cardiac dysfunctions [18, 19]. Chelation therapy helps balance the amount of iron accumulated in blood transfusions by increasing iron discharge in urine and/or feces with chelators [20].

Due to the promising results of using iron chelators both individually and combined for the treatment of iron overload, iron chelators have become increasingly important in medicine over recent years. Chelation therapy has a long history dating back to Ferdinand Mans working on the synthesis of ethylenediaminetetraacetic acid in the early 1930s [6, 21].

Iron chelation therapy follows several goals. Prevention therapy balances iron absorption from a blood transfusion with iron excretion to ensure healthy levels of body iron. Rescue therapy should be performed before harmful amounts of iron have accrued. Emergency therapy is performed in the cases of heart failure that involves immediate intervention and normally entails the modification or intensification of the treatment. Dose adjustment therapy involves the adjustment of dosing and treatment regimens in response to changing conditions. Chelation therapy should be received on a daily basis to be successful. This requires a deep commitment to the chelation protocol [22, 23].

Iron-specific chelators include deferoxamine (DFO), deferiprone (DFP), and deferasirox (DFX). They each have their own set of benefits and drawbacks [24]. Since 1987, the oral chelator DFP has been licensed for human consumption [25]. Several trials have shown the safety and effectiveness of chelation therapy in iron overload in patients with beta-thalassemia (*β*-thalassemia) major (TM) who receive chronic transfusions. Several factors, such as the severity of iron overload, cure duration, and final costs of treatment, should be considered to determine the best chelation therapy for a specific clinical condition [26, 27]. This review aimed to summarize the data on iron chelation therapy and its purpose, different types of iron chelators and their advantages and disadvantages, and toxicity and side effects of these iron chelators, compare strong and weak points of different combination therapies, and discuss the future prospects of iron overload treatments.

## 2. Methods

This study was conducted by reviewing and summarizing the data on iron chelation therapy in iron overload. The searched keywords included “Iron Overload,” “Chelators,” “Deferoxamine (DFO),” “Deferiprone (DFP),” “Deferasirox (DFX),” and “Combination Therapy.” The keywords were searched in English in the reported and published articles of journals up to September 2021 using several online databases, including Google Scholar, PubMed, Scopus, Institute for Scientific Information, Web of Science, ResearchGate, and ScienceDirect. The reference lists of the extracted articles were searched manually.

## 3. Iron Chelation Therapy

Iron-chelation therapy has been successfully used from the early 1980s for the treatment of iron overload in thalassemia intermedia (TI), other iron-loaded nontransfusion-dependent thalassemias (IL-NTDT), and other similar conditions [28]. Iron chelation therapy stays a pillar of NTDT management and is one of the most practical ways of reducing mortality and morbidity [29]. Some restrictions related to dose, cost, and toxicity are not deniable. The usage of chelation therapy for a rise in iron excretion, inhibition of iron absorption, and iron redistribution; chosen diets with lower and less absorbable forms of iron; usage of regulators of divalent metal transporter 1, ferroportin, and other proteins of iron metabolism involved in the transport of iron; activators of fetal hemoglobin production; and combinations of such treatments can become accessible for the future management of TI, other IL-NTDT, and hereditary hemochromatosis [28].

The main purpose of chelation therapy is the entire elimination and prevention of iron overload. Chelation can clear away the surplus iron load and keep iron at natural levels. The treatment of patients with iron chelators can decrease the toxic effect of iron overload. Iron chelators enter cells, bind free iron, and remove it from the body. In young patients with severe chronic anemia, iron chelation therapy is highly accepted; however, for older patients suffering from the same illness, the same treatment is doubtful due to myelodysplastic syndromes. Some aspects of hematological disorders, such as the stimulation of platelet production, inhibition of leukemic cell proliferation, and induction of their differentiation, can also be benefited with iron chelator activity [30].

An iron chelator should reach the chemical requirement. For meeting the requirement, the chelator should be selective for iron and should not be transformed into nonchelating metabolites once it is in the organism. There should be a rapid kinetic exchange of iron between chelator and endogenous ligands. The complexes made by the chelator and iron should have appropriate redox and higher steadiness than complexes made by iron and endogenous ligands. It should also have the biomedical requirement, which is the low toxicity of the formed complexes and the chelating agent, good abdominal absorption, and good bioavailability of the chelator in entering the target cell [31].

Different iron chelators can be administered in the treatment of iron overload. The DFP, DFX, and DFO are important iron chelators, each of which has its advantages and disadvantages [32, 33]. The DFO is a nontoxic parenteral iron chelator approved by the clinic and fruitful for long-term iron chelation therapy. The DFP is an oral iron chelator and a great option for patients who have not gotten results from DFO or DFX. The DFX is an oral tridentate iron-chelating agent which attaches iron in a 2 : 1 ratio [32, 34, 35].

There are numerous factors to consider while choosing proper chelation, such as the cost of medication, treatment period, how to use, and the proliferation of tumor cells [32]. Clearing away all surplus stored iron and maintaining normal iron stores using DFP and chosen combinations with DFO are helpful for different categories of thalassemia patients. If patients cannot tolerate DFO and DFP, DFX can be used [36]. The oral options of iron chelators are associated with improved adherence. The new film-coated tablet (FCT) formulation of DFX can be governed in a single step without preparation and with less stringent food restrictions and is more palatable, with fewer gastrointestinal side effects that might be experienced with the original oral suspension formulation. Therefore, these therapeutic attributes can reduce the complications resulting from iron overload. Additionally, the usage of combination and consecutive chelating agents can enhance efficiency and decrease the incidence of adverse events [37].

**Figure 1:**
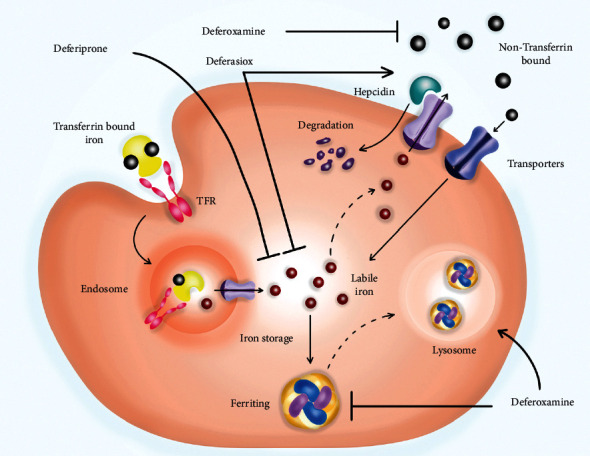
Deferoxamine, deferiprone, and deferasirox mechanism of action in the management of iron overload. Deferoxamine binds to nontransferrin bound iron or to iron found in ferritin forming a molecule which is later excreted via the kidneys. Deferoxamine also promotes ferritin degradation in lysosomes. Deferiprone and deferasirox chelate cytosolic labile iron. Besides, deferasirox can increase the levels of hepcidin that results in the degradation of ferroportin. TFR, transferrin receptor.

Averting or clearing away cardiac iron loading is one of the main purposes of iron chelation therapy. Designing a chelator that can cross cellular membranes, is orally active, and is adequate to take iron from the heart, brain, or liver to spare essential iron is challenging for the future. Several oral iron chelators, including an alpha-ketohydroxypyridine analog of DFP, 1‐(N‐acetyl‐6‐aminohexyl)‐3‐hydroxy‐2‐methylpyridin‐4‐one, and a novel oral once-daily iron chelator (FBS0701100), are under development [38] (Figure 1).

## 4. Different Types of Iron Chelators

### 4.1. Deferoxamine (DFO)

The DFO is the first drug used to treat excess iron and has parenteral administration. Since oral DFO absorption is low, it is administered intravenously or intramuscularly to be beneficial [30, 31, 37, 39]; this is why only a fraction of patients consent to thalassemia treatment with DFO. This administration route is one of the most considerable limitations of the treatment with DFO [31]. The need to use DFO parenterally is why numerous patients refuse to use this drug, thereby reducing its potential benefit [31].

Daily subcutaneous administration of DFO over 8 to 12 hours is recommended to treat chronic iron overload in patients over 3 years [37]. The DFO is initiated when ferritin exceeds 1000 ng/mL. This drug has a significant association with a reduction in serum ferritin levels, hepatic iron, and cardiac and endocrine complications in patients with iron overload [34, 38]. However, while using iron chelator, some patients experienced cardiac disorders, which is the leading cause of death in the third decade of life. Since these patients also have multiorgan iron overload and infective complications, particularly hepatitis C as a result of repeated blood transfusions, heart transplantation is a limited therapeutic choice [34, 40]. This treatment requires time obligation and compliance of the patients. Since the treatment is intricate, there is a great rate of no adherence, making adherence an important challenge [30, 38].

The DFO is given by a subcutaneous infusion with a mobile pump for 5–7 nights/week or with a 24-hour infusion into a patient's vein. The appropriate dose of DFO for adult patients is within 1–2 g and about 20–40 mg/kg for pediatrics [30]. A study indicated that the best drug for the treatment of transfusion iron overload is DFO, especially when used continuously instead of intermittently [34]. Another study suggests that DFO was more effective than DFP in the treatment of TM and reduction of the ferritin level [41]. In *β*-thalassemic patients, iron chelation with DFO has reduced and deferred liver disease, diabetes, endocrine failure, and normalized growth and prevented cardiac insufficiency [42].

The adverse events related to DFO are growth retardation, skin reactions, ocular, auditory, allergic reactions, and bone abnormalities. In addition, pulmonary and neurological disorders were observed at high doses [38]. Moreover, the intense harm of DFO is its mode of administration via a portable injection pump daily. Adoption and adherence to daily infusions of DFO infusions are important restricting agents in the achievement of treatment objectives [43]. The DFO reduces blood cell adherence to endothelial cerebral venules [44].

The early trials of DFO chelation treatment did not affect the progression of progressive endocrine dysfunction [45, 46]. Only severe chelation treatment with a two-drug combination (i.e., DFO and DFP) has improved endocrine complications outcomes [47]. However, this intense chelation is usually only used in an emergency and for a limited time, as it might have a detrimental influence on therapeutic compliance and drug-related side events [45]. Although iron can induce kidney toxicity, it is also an important cofactor in renal prostaglandin production, and a lack of prostaglandins as a result of iron deficiency can lower the glomerular perfusion rate and glomerular filtration rate (GFR). Furthermore, it is conceivable that the lower GFR observed with DFO and DFX is due to so-called “relative iron depletion” [48]. Acute renal failure induced by toxic tubular injury has been documented in individuals taking DFO, albeit uncommon [48]. When DFO treatment is contraindicated or insufficient, DFX is the only iron chelator expressly authorized in transfusion-dependent *β*-thalassemia (TDT) and NTDT individuals 10 years and older [49] (Table 1).

### 4.2. Deferiprone (DFP)

The DFP was the first available oral iron chelator. It was synthesized in 1981 at the University of Essex, England, and approved by Food and Drug Administration (FDA) in 2011 for the treatment of patients suffering from transfusional iron overload due to thalassemia syndrome when the response to chelation therapy is inadequate [31]. The DFP is an active hydroxypyridinone used in humans for the first time in 1987 [50] and is currently used in over 50 countries [31]. It is a bidentate ligand binding to iron in a 3 : 1 ratio and has a half-life of 2–3 hours; therefore, it should be given to patients three times a day [38, 51].

The DFP is most likely effective in cardiac iron clearance [52]. It reduces red blood cells membrane oxidative damage and the formation of lipid oxidation products [44]. Gastrointestinal symptoms and agranulocytosis are two of the most common adverse events occurring in patients taking DFP [37, 53]. Agranulocytosis is defined as an absolute neutrophil count below 500/*µ*L [51]. The neutrophil count should be monitored weekly due to the risk of agranulocytosis, and approximately 10% of patients stop using the drug permanently due to its side effects [37, 53]. The DFP therapy increases urine iron excretion. Its amount is associated with the total daily dose of the drug within the range of 25–100 mg/kg daily [54–56]. The recommended dose for this iron chelator is 75 mg/kg in three divided doses per day, and the maximum recommended dose is 100 mg/kg per day [32]. In pediatric patients, DFP could be sufficient and effective at a similar dosage that is used in adult patients, and there is not any significant augmentation in the risk of agranulocytosis and other reported relevant side effects, although caution is most recommended in integrated treatment if two chelators DFO and DFP or DFX and DFP are applied at the same time [51]. A study indicated that DFP liquid formulation could be considered secure in children suffering from transfusion-induced iron overload. All of the nine patients involved in the study tolerated the liquid formulation well. Moreover, they did not experience vomiting, gastrointestinal discomfort, or nausea [57].

The DFP is predesignated to improve adherence. Despite the fact that total iron excretion using DFP is a bit less, compared to that of DFO, DFP cardioprotective effect is better than DFO since DFP is able to penetrate the cell membrane [43]. The DFP is very efficient in eliminating extra iron from the organs, especially the heart [30]. It is demonstrated that DFP improves left and right ventricular ejection fraction [58–61]. A retrospective survey demonstrated that the administration of DFP over time improves the cardiac ejection fraction better than DFO [62]. Additionally, in studies based on epidemiological data, mortality and morbidity in cardiac events were lower in individuals taking DFP rather than DFO [34].

Furthermore, oral DFP was observed with superior effects on SCD morbidity [63]. In the deficiency of chelation therapy, pediatrics show signs of iron overload after about 5–10 transfusions; nevertheless, the primary initiation of DFP treatment at 50 mg/kg per day can dramatically postpone iron overload [64]. The DFP can be used in pediatrics mostly after DFO as a second-line chelator. This made young children use parenteral chelators and prevented them from using oral chelators [51]. A study aimed to evaluate the most effective iron chelator in the reduction of hepatic and cardiac iron assessed through T2∗ magnetic resonance imaging (MRI). The results for the most efficient iron chelator in reducing myocardial iron and hepatic iron were DFP and DFO, respectively [65]. The DFP is used in the treatment of blood disorders, such as thalassemia, and its effectiveness was assessed in some studies [41] (Table 2).

### 4.3. Deferasirox (DFX)

The DFX gained clinical use as the second oral iron chelator and obtained approval from the FDA in 2005. It is a successful oral iron chelator with a long half-life. The 24-hour chelation will be provided using it once a day. It is a tridentate iron chelator, which binds to iron in a 2 : 1 ratio [31, 66]. The DFX is approved in numerous countries for the therapy of chronic iron overload. The safety and efficacy of DFX have been assessed in patients suffering from *β*-thalassemia and in other kinds of anemias, including Diamond-Blackfan anemia, SCD, myelodysplastic syndrome, and aplastic anemia. Chelation therapy with DFX is very advantageous due to its once-daily formulation [38, 67, 68]; however, it is considered to be very expensive, and many cannot afford it [69].

There are two different once-daily formulations available for DFX, namely DFX FCT, which is a newer formulation approved in 2015, and DFX dispersible tablet (DT), which is the original formulation [70]. One year of therapy using DFX DT illustrated a dose-dependent decrease in serum ferritin and liver iron concentration in patients with transfusion-dependent SCD and *β*-thalassemia [37]. The affection of DFX DT has been determined by testing a large group of patients with thalassemia, myelodysplastic syndromes, and SCD [71–76]. The highest dose approved for this iron chelator is 30 mg/kg per day in numerous countries. Nevertheless, some patients need more than 30 mg/kg per day to reach their therapeutic goals. A study evaluated the safety and efficacy of DFX doses of more than 30 mg/kg per day in patients suffering from transfusion-dependent anemias, such as myelodysplastic syndrome, SCD, and *β*-thalassemia. The safety profile was very notable. All the adverse events were the same as the adverse events in patients who received lower doses of DFX. Furthermore, the studies showed that the escalation of these iron chelators' doses to more than 30 mg/kg per day lowers iron burden [77]. Based on recent research, DFX about 20–30 mg/kg per day can protect or improve hepatic iron in patients more than DFO [34]. The DFX reduces liver iron in TI and *β*-TM and raises iron excretion [28].

A large multicenter study assessed the long-term efficacy and safety of DFX in children with SCD and TDT. Serum ferritin levels had a marked decrease in 3 years of therapy, and there was a slight elevation in the amount of serum creatinine which was considered to be related to the adverse effects of DFX [78]. The DFO and DFX are effective and well-tolerated in lowering serum ferritin levels in patients with *β*-TM. However, DFX is highly recommended due to its convenience and adherence [79]. A recent study by charity et al. [80] compared adherence rates between the formulations of DFX and described associations between adherence to chelation therapy and changes in hematological parameters among patients with SCD and *β*-thalassemia. The first formulation of DFX, a DT for oral suspension called Exjade®, was associated with adherence challenges due to complaints of poor taste and side effects, such as abdominal discomfort. A new FCT formulation called Jadenu® was subsequently developed to overcome these challenges. Accordingly, there was a significant improvement in adherence to iron chelation therapy when patients transitioned from DFX DT to DFX FCT.

The most frequent adverse events are skin rashes, gastrointestinal side effects, and an increase in serum creatinine. The DFX FCT can raise adherence more than the original formulation. Furthermore, DFX FCT has fewer gastrointestinal adverse effects, compared to the original formulation [37, 81]. The DFX has been demonstrated to be efficient in chelating iron from the heart and liver, preserving heart function, and reversing hepatic fibrosis [82–84]. Monotherapy with DFX has been linked to a gradual worsening in glycemic control [85–87]. The DFX treatment is superior to other chelators. Over the course of 5 years of medication, it has consistently reduced the serum ferritin level and liver iron concentration, which is a proxy for total body iron [88]. Low-dose DFX was used in transplant thalassemia patients. It had a manageable adverse effect profile, and the rate of therapy cessation was low over a long period [89, 90]. Furthermore, the once-daily formulation and oral mode of administration of DFX define higher acceptability of chronic chelation treatment and an improvement in chelation therapy compliance [90].

**Figure 2:**
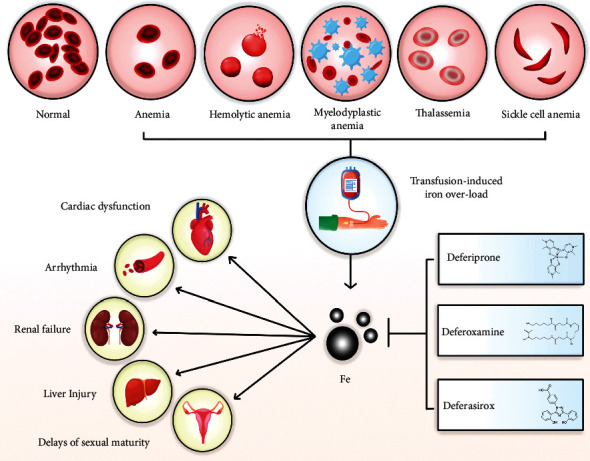
Deferoxamine, deferiprone, and deferasirox effects on transfusion-induced iron overload. Patients with aplastic anemia, hemolytic anemia, myelodysplastic anemia, thalassemia, and sickle cell anemia become transfusion dependent. Iron toxicity leads to free radical production, which causes severe side effects, including cardiac dysfunction, arrhythmia, renal failure, kidney damage, and delays in sexual maturity. Iron chelators can enter cells, bind free iron, and remove it from the body, thus inhibiting iron toxicity.

The DFX-based iron chelation treatment can help prevent endocrinopathy [91, 92]. During long-term DFX medication in *β*-TM patients, a stabilizing impact on endocrine abnormalities and a low risk of incident endocrine illness in a natural clinical practice environment were observed [90]. Fanconi syndrome [93], characterized by hypokalemia, hypophosphatemia, hypercalciuria, metabolic acidosis, hyperaminoaciduria, and hyperuricosuria, is a widespread malfunction of proximal tubular cells caused by DFX [48]. Chelation could be started with low-dose DFX before 2 years of age, with close monitoring of motor and cognitive development as there is little chance to reverse endocrine overload [47]. However, the specific method through which DFX affects the bone is unknown [90]. The DFX, but not other chelators, caused favorable changes in bone mineral density and a decrease in osteoporosis prevalence after 5 years of treatment [92]. The most common renal adverse effect of iron chelators (mostly DFX) is a slight reduction in GFR, which is typically transitory and reversible by decreasing the medication dose [94] (Table 3 and Figure 2).

## 5. Toxicity and Side Effects

When the iron-binding capacity of transferrin and ferritin is exceeded, iron can produce damaging free radicals [95, 96] and lead to delays in sexual maturity [97], impotence and infertility [12], cardiac dysfunctions such as arrhythmia and cardiomyopathy, hemosiderosis [2], liver cirrhosis [13], liver cancer, hepatitis [13], and metabolism dysfunctions such as diabetes, hypogonadism, thyroid and parathyroid disorders, lower level of adrenal glands [14], inherited hemoglobinopathies, thalassemia, and SCD [98–101]. Iron overload toxicity from chronic transfusions and increased gastrointestinal iron absorption result in progressive multiorgan damage and corresponding growth in morbidity and mortality, which in most cases is directly related to the stage of excess iron load [98, 99, 101–105]. Congestive cardiac failure due to cardiac iron overload toxicity has been the basic result of death in iron overloaded thalassemia cases [104, 106]. The iron overload of IL-NTDT can progressively result in cardiomyopathy, hepatic fibrosis, cirrhosis, hepatocellular carcinoma, and diabetes [105, 107, 108]. Genetic, dietary, immunological, and other factors affect the iron overload rate and associated toxicity in IL-NTDT and TM [105, 107, 109]. Moreover, as previously mentioned, iron chelators used for lowering iron load in the body can also cause side effects.

### 5.1. Deferoxamine Toxicity

The DFO treatment has some side effects, such as visual, auditory neurotoxicity, hearing and vision loss, abdominal pain, skeletal alterations, growth delay, and respiratory distress syndrome that could occur in the treatment course of acute iron poisoning and in irritation at the infusion site, diarrhea, nausea, anaphylaxis, vomiting, hypotension, increasing blood pressure in lungs, and the risk of infection of *Vibrio* and *Yersinia* [30, 31, 110, 111]. These microorganisms are known as siderophilic. The liberation of iron from the stores in the body is a reason these bacteria thrive in patients on chelation [112].

### 5.2. Deferasirox Toxicity

The DFX is mostly tolerated, and its most common side effects are skin rashes and gastrointestinal disorders [66]. Serum ferritin levels persistently over 2500 mg/L and liver iron over 15 mg/g dry weight were related to cardiac disease in patients receiving DFX. Adverse events related to DFX that were detected in approximately 10% of patients include rash, diarrhea, nausea, and abdominal pain. Kidney and liver functions could become irregular. It is recommended to test their function monthly. In high-risk patients, more frequent testing should be considered. The organization of zinc polymeric complexes could have a role in kidney complication's origin. Other side effects are fever or allergic reactions, auditory or visual defects, metabolic acidosis, hypophosphatemia, hypokalemia, cytopenia, growth delay, hemorrhage, bone defects, sporadic proteinuria, gastrointestinal problems, and slight serum creatinine level enhancements [3, 30, 31, 113–119]. In addition, hematological toxicities, such as the reduction of erythropoiesis, myocardial toxicities, such as focal degeneration, inflammation, and myocarditis at the highest dose of 100 mg/kg, were reported.

Side effects depend on the dose and reverse with discontinuing drug consumption; however, the solution causes insufficient iron chelation. Due to these points, the interpersonal variability of the plasma concentration of the drug helps the clinical administration of DFX dosage. The decision of the treatment of patients based on their own characteristics contributes to enhancing efficacy and minimizing the toxicity of DFX therapy [114]. Another study reported that most DFX-related adverse events were mild to moderate. Furthermore, these adverse events were resolved without the discontinuation of treatment [67]. In a patient who received a 20–40 mg/kg dose of DFX, there was maculopapular erythematous skin rash on the trunk, arms, and legs, which tended to happen around 1 week after the onset of high-dose therapy of 40 mg/kg. Other adverse effects included increased transient transaminases, diarrhea, moderate nausea, mild abdominal pain, and headache. Doses of 20 and 30 mg/kg produced stable or decreasing liver iron content over 1 year of therapy; nevertheless, doses of 5 and 10 mg/kg were too low to produce this effect [120–122].

In three studies, adverse events were compared between the DFO and DFX groups. After pooling data, significantly higher risk ratios were acquired in the DFX treatment group in comparison to those of the DFO treatment group in alanine transaminase and serum creatinine with low heterogeneity. However, alanine transaminase and serum creatinine rates recovered following drug discontinuation. It can also be a false-positive outcome due to the short study period and the limited number of cases. Rashes, gastrointestinal symptoms, and severe adverse events were more frequent in the DFX treatment group than in the DFO treatment group. No significant differences were observed between the DFO and DFX treatment groups which could be due to inconsistent or incomplete reporting and decreased sample size. The outcomes demonstrated that the risk ratio of the DFX treatment group was higher than the placebo group, with slight heterogeneity in the levels of rashes, abdominal events, and severe adverse events. Nevertheless, the aforementioned outcomes were not statistically significant [75, 76, 123]. Another study indicated that adverse events, such as gastrointestinal events, seemed to be more frequent with DFX [124].

A study reported that DFP with low-dose DFX was associated with side effects such as arthralgia, nausea, vomiting, headaches, visual disturbances, and death. During DFP therapy, the body's iron burden increased in stages that put patients at risk of glucose intolerance, cardiac disease, and premature death.

### 5.3. Deferiprone Toxicity

The significant side effects of DFP are agranulocytosis and neutropenia that occur in approximately 1.7% of the cases [125, 126], and less frequent severe adverse events include gastrointestinal symptoms, arthropathy, musculoskeletal pains, transient changes in liver enzymes, zinc deficiency [31, 127], nausea, abdominal pain, vomiting, and arthralgia [3, 127]. The mechanism through which DFP causes agranulocytosis has been unknown to date. Neutropenia and agranulocytosis are usually resolved with the suspension of the DFP treatment although some cases of fatal agranulocytosis were observed in postmarketing monitoring. Therefore, cases receiving DFP treatment should be strictly followed up for neutropenia and agranulocytosis by taking a complete blood count weekly [128]. Nevertheless, it is reported that there were no severe, serious, or unexpected adverse events in the early start of therapy with DFP in young children newly diagnosed with TDT [64].

## 6. Combined Therapy with More Than One Chelator

Combined therapy is an alternative way of the adjustment of iron levels in patients who were not able to respond completely to monotherapy [110]. The treatment of iron overload using more than one iron chelator brings about a number of benefits, including better accessibility to various iron pools, better control on nontransferrin bound to iron, better tolerability, improved compliance, reduced myocardial iron, and improved cardiac dysfunction in iron-overload cardiomyopathy. By decreasing the dosage of both chelators, not only the adverse effects of these chelators will be decreased but also at the same time, they will manifest high chelation efficiency [31, 34, 129]. It was also observed that the amount of iron removed via combined therapy was more than the total iron eliminated individually by each chelator [31]. Therefore, researchers started using some of these chelating agents in the patient's regimen at the same time that was effective, especially DFO and DFP, which led to promising results in different studies [110].

### 6.1. Deferoxamine with Deferasirox

The combination of DFO with DFX might have better results because their toxicity profiles do not overlap, and they can reach the intracellular iron pool quickly. Moreover, they both have a low molecular weight [130, 131]. For the evaluation of the effectiveness of the combination form, the present study previously reviewed some articles. In a recent study, nine patients with TDT used DFX at a dose of 20–40 mg/kg per day and DFO at a dose of 18–40 mg/kg per day simultaneously for 3–6 days per week. After 12 months, eight patients were observed with dropped serum ferritin levels and decreased liver iron concentrations. The combination of these two chelating agents proved to be safe and without major toxicities [132]. Another study included 62 patients with TM that were assessed for the effectiveness of DFX monotherapy, compared to that of DFX with DFO combined therapy. In the aforementioned study, 30 mg/kg oral DFX daily or 50 mg/kg DFO plus 30 mg/kg DFX daily were given to patients 5 days a week in a 1 : 1 ratio. Both groups received therapy for a total of 12 months. Myocardial T2∗ values had a marked rise in the group with combined therapy. Serum ferritin decreased in both groups, and liver T2∗ values did not have a significant change in both groups. Therefore, it can be observed that using combined therapy for TM patients with DFO and DFX is more effective than only using DFO [133]. A case study reported that using the combination of DFX and DFO in *β*-TM patients had a beneficial effect on the liver and heart hemosiderosis. The treatment was well tolerated, with no reported side effects [134]. Furthermore, a combination of DFX and DFO was related to better serum ferritin levels than the monotherapy of DFO, DFX, DFO plus silymarin, and DFP plus DFO [63].

### 6.2. Deferoxamine with Deferiprone

The DFP and DFO chelation together decreases serum ferritin, liver iron, and myocardial siderosis, enhances cardiac function, reverses and avoids endocrine disorders, lowers cardiac mortality, and improves survival, according to long-term experience [40, 129]. This combined therapy was also associated with better left ventricular ejection fraction, low risk of adverse event and mortality, and reduced serum ferritin than DFO therapy alone [63]. The DFO binds liver iron more efficiently, resulting in biliary iron excretion; however, DFP binds parenchymal iron more broadly [127].

For the assessment of the impact of combined therapy in patients with different diseases, some studies are evaluated. The toxicity of iron in TI is usually obvious at 30–40 years of age and in TM at 10 years of age. Subcutaneous DFO, oral DFP, and their combination were productively used for iron toxicity therapy in TM and TI patients that resulted in a decrease in the patient outcome measures, the elimination of the whole addition toxic iron, and the prevention of cardiac, liver, and other important organs' damage [28, 36]. In 1998, it was observed that some transfusion-dependent TM patients did not reach the negative iron balance with 75 mg/kg per day DFP. Therefore, they were proposed combined therapy with DFP and DFO [31]. In thalassemia children, combination therapy of daily oral DFP and subcutaneous DFO twice weekly is a safe and effective alternative to chelation monotherapy [135]. Patients with homeostatic iron regulator hemochromatosis and low erythrocytapheresis tolerance can benefit from the addition of iron chelators [136].

In patients with TDT presenting cardiac problems, combined chelation therapy with high-dose DFP and DFO is vital. Combined therapy in these patients was successful in the improvement of survival and cardiac function. In all patients, the arrhythmia was reverted to sinus rhythm [137]. In a clinical experiment on 36 patients with *β*-TM, DFP was given orally for 6 days per week at a total daily dose of 60 mg/kg, and DFO was given subcutaneously for 4–6 days per week at a total daily dose of 40–50 mg/kg. Serum ferritin and 24-hour urinary iron excretion levels were used to determine the effectiveness of the combined therapy. The findings of the aforementioned study indicated that combining DFP and DFO with iron chelation therapy resulted in a satisfactory reduction of serum ferritin with no major side effects [138].

In a recent study, DivakarJose et al. [139] assessed the efficacy and safety of dual oral iron chelation therapy (i.e., DFO and DFX) in decreasing iron overload status. They used serum ferritin, liver, and cardiac MRI as indicators in TDT children. A total of 21 thalassemic children with a mean age of 7.8 years were enrolled. It was concluded that combined oral chelation with DFP and DFX significantly decreased the serum ferritin level in children with severe iron overload. The drugs were tolerated well without any serious adverse effects. Other studies have also proven the effectiveness of combined DFO and DFP therapy in iron overloaded *β*-TM [140–146]. Synergistic effects were observed in a significant number of patients, meaning that the total amount of iron removed in combined therapy was greater than the sum of the amounts removed individually by each chelator [31, 129]. In line with the aforementioned findings, DFO and DFP combination is one of the most effective medications that have been used for chelation therapy in different illnesses.

### 6.3. Deferasirox with Deferiprone

Among the three iron chelators in use, DFP is considered to have the best efficiency in reducing cardiac iron overload, and DFX is regarded as the effective total body iron chelator. Therefore, the DFX and DFP combination might be beneficial by making chelation agents cautiously available in-patient blood circulation. This combination also enhances iron excretion and reduces free labile iron. Another advantage is that the toxicity profile of these two agents is nonoverlapped. The hypothesis is that thrice-daily use of DFP and once-daily use of DFX will ensure that iron chelator agents are exposed, and toxic iron species are suppressed. Therefore, the end-organ damage will be limited. A study on 36 pediatric patients showed successful results in terms of safety, efficiency, and patient compliance to combination therapy [147, 148]. In terms of frequency, a study suggested that the effective way to reduce serum ferritin and liver iron levels in patients chelated on DFX or DFP alone is a combined therapy, in which DFP is given every day, and DFX is given 2–4 days each week [54].

Based on previous studies, DFP plus DFX combined therapy can be considered to alleviate cardiac and liver iron overload in heavily overloaded *β*-TM patients. The improved values of heart and liver T2∗ MRI were observed after the combined therapy of DFP plus DFX [149]. In a case report, a 25-year-old woman with TM received blood transfusion over the past decades. This patient's response to DFP plus DFX therapy was so significant that it led to hypoferritinemia, and a normal cardiac index was reported in the follow-up imaging. The combined therapy of DFP plus DFX seems promising for patients who do not comply with DFX and patients with developed high cardiac siderosis [150]. The DFX plasma levels increase in combination therapy of DFP plus DFX; consequently, the reduced dosage of DFX can be considered in combined therapy [151].

**Figure 3:**
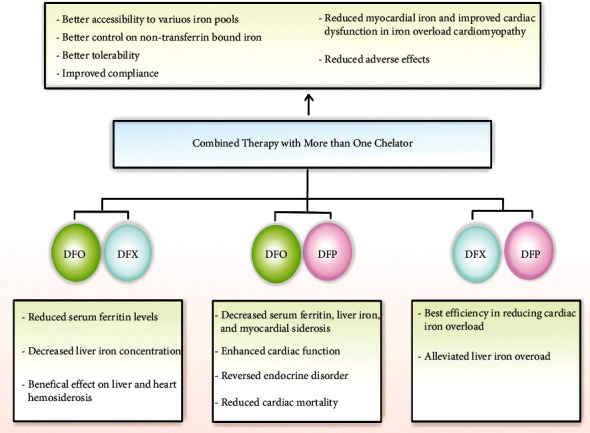
Combination therapy with deferoxamine, deferiprone, and deferasirox.

The DFP with DFX combination was superior in the improvement of cardiac T2∗, medication compliance, and patient satisfaction while causing no additional adverse events [152]. Altogether, the oral combination of DFP and DFX is a potentially promising approach, especially in patients who are heavily overloaded and do not respond well to monotherapy [147, 148]. The DFP and DFO versus DFP and DFX was compared in terms of safety, effectiveness, compliance, treatment satisfaction, and quality of life. Both iron chelation combination treatment regimens were equally effective in the reduction of iron overload and improvement of the quality of life [152] (Table 4 and Figure 3).

### 6.4. Future Perspectives

In a recent study, Zhang et al. [153] designed N-hydroxyalkyl-substituted DFP as a kind of iron-chelating agent for Parkinson's disease chelation therapy strategy. The chelator both met Lipinski's rule for blood-brain barrier permeability and ensured the iron affinity. Their results demonstrated that firstly the pFe3+ value of N-hydroxyalkyl substituted DFP is within 19.20 to 19.36, which is comparable to that of clinical DFP. Secondly, N-hydroxyalkyl-substituted DFP also possesses a similar radical scavenging ability in comparison to DFP. Thirdly, the N-hydroxyalkyl-substituted DFP exhibits extremely low cytotoxicity and an excellent H_2_O_2_-induced oxidative stress protection effect. The aforementioned results indicated that N-hydroxyalkyl-substituted DFP has potential application prospects as a chelating agent for Parkinson's disease chelation therapy strategy.

In another recent study, Komoto et al. [154] revealed the first success of the design of polymeric DFO for the enhancement of iron chelation cancer therapy. They developed polymeric DFO by the covalent conjugation of DFO to poly(ethylene glycol)poly(aspartic acid) (PEGPAsp) block copolymers. Subsequent to intravenous administration, the polymeric DFO showed a marked increase in blood retention and tumor accumulation in subcutaneous tumor models. Consequently, polymeric DFO showed the significant suppression of tumor growth, compared to free DFO. However, clinical trials are needed to further prove the efficacy of these novel iron chelators. A log P-guided investigation of 20 hydroxpyridinones resulted in the identification of CN128. A study by Chen et al. [155] showed that the Fe(III) affinity and metal selectivity of CN128 are similar to those of DFP, the log *p* value is more lipophilic, and its iron scavenging ability is superior. It was concluded that CN128 was safe in a range of toxicity assessments and is currently used in clinical trials for the treatment of *β*-thalassemia after regular blood transfusion.

In another recent study, Bailey et al. [156] presented a novel and promising mechanism for therapeutic iron chelation. Accordingly, they concluded that PBT434, currently being developed for the treatment of Parkinson's disease and multiple system atrophy, could both chelate interstitial iron and inhibit the reuptake of iron by the endothelial cells of the blood-brain barrier and inhibit its uptake by the other cells of the neurovascular unit. Recently, in an in vitro study, Calabrese et al. [157] reported that DFX-dependent iron chelation could enhance mitochondrial dysfunction and restore p53 signaling by the stabilization of p53 family members in leukemic cells. Clinical trials are required to prove these chelators' effectiveness and report possible side effects.

On the other hand, some chelators have shown little or no efficacy and several side effects. Chelators containing -H, mono-, or dihydroxyalkyl and diethoxyethyl 1-substituents caused little or no increase in iron (59Fe) excretion by the intraperitoneal or oral routes. In vitro studies using hemosiderin and ferritin have demonstrated that equivalent iron release occurred with both groups of chelators independent of their in vivo effects. In most cases, there was a positive correlation between the lipophilicity and acute or subacute toxicity of these chelators in rats. The most toxic chelator in the chronic toxicity investigation in rats was the lipophilic 1,2-diethyl-3-hydroxypyrid-4-one. Altogether, the oral effectiveness in increasing iron excretion by these chelators in animals is not related to their lipophilicity or their ability to mobilize polynuclear iron in vitro but rather to other features possibly related to their rates of excretion and biotransformation [158].

## 7. Conclusion

Iron chelation therapy has been used to treat diseases accompanying iron overload. Keeping a safe amount of iron in the body by balancing iron intake with iron excretion is the main purpose of chelation therapy. Using chelating agents tends to be beneficial, and patients who have taken these medications have shown positive outcomes. It has been discovered that the simultaneous use of two chelating agents produced stronger outcomes than monotherapy. Despite evidence scarcity, it is clear that iron chelation therapy is useful in the treatment of iron overload and improvement of overall survival. Finally, in the near future, further studies will demonstrate complete information and knowledge of iron chelation therapy.

## Figures and Tables

**Table 1:** Summary of the findings on deferoxamine.

Author (year)	Property	Route	Dose	Schedule	Side effects	Reference
Taher et al. (2018)	Deferoxamine	Subcutaneous or intravenous	30–60 mg/kg per day	8–12 hours, 5–7 days per week	Ocular and auditory symptoms, bone-growth retardation, local reactions, and allergy	[49]
Poggiali et al. (2012)	Deferoxamine	Subcutaneous and intravenous	20–40 mg/kg/day	Over 8–24 hours, 5 days/week	Local skin reactions, visual and auditory allergic reactions, growth retardation, bone abnormalities, and pulmonary and neurological disorders at high doses	[38]
Crisponi et al. (2019)	Deferoxamine	Intravenous and subcutaneous	20 and 60 mg/kg/day	8–10 hours a day, 5–7 days a week	Growth delay, skeletal alterations, visual and auditory disturbances, and respiratory distress syndrome	[31]
Shah et al. (2017)	Deferoxamine	Subcutaneous, intravenous, or intramuscular	20–40 mg/kg/day	Daily subcutaneous administration over 8–12 hours	Injection-site reactions and visual and auditory disorders, particularly in older patients (most cases were reversible)	[37]
Hershko et al. (2005)	Deferoxamine	Intravenous	50 mg/kg/day	5 days per week	—	[43]
Fibach and Rachmilewitz (2017)	Deferoxamine	Subcutaneous or intravenous	30–60 mg/kg per day	8–12 hours, 5–7 days per week	Ocular and auditory symptoms, bone-growth retardation, local reactions, and allergy	[30]

**Table 2:** Summary of the findings on deferiprone.

Author (year)	Property	Route	Dose	Schedule	Side effects	Reference
Poggiali et al. (2012)	Deferiprone	Oral tablet or solution	75–100 mg/kg/day	In three divided doses daily	Gastrointestinal symptoms, agranulocytosis, neutropenia, arthralgia, and elevated liver enzymes	[38]
Maggio et al. (2002)	Deferiprone	Oral	75 mg/kg	Divided into three daily doses administered as 500 mg pills every 8 hours	Hypertransamin/asemia and leukocytopenia	[58]
Elalfy et al. (2018)	Deferiprone	Oral	16.6–75 mg/kg	Per day	Neutropenia and agranulocytosis	[64]

**Table 3:** Summary of the findings on deferasirox.

Author (year)	Property	Route	Dose	Schedule	Side effects	Reference
Antmen et al. (2018)	Deferasirox	Oral	26.4 ± 6.1 mg/kg/day in TDT patients	N/A	Increased hepatic enzyme, renal tubular disorder, increased blood creatinine, abdominal pain, and proteinuria	[78]
Cappellini et al. (2006)	Deferasirox	Oral	5, 10, 20, and 30 mg/kg/day based on liver iron concentration	Once a day	Increased serum creatinine, elevated alanine aminotransferase, auditory disorders, cataracts or lenticular opacities, and gastrointestinal events including abdominal pain, nausea, vomiting, diarrhea, constipation, and skin rash	[76]
Galanello et al. (2006)	Deferasirox	Oral	10 mg/kg/day	Once a day	Nausea and skin rash	[71]
Hassan and Atef Tolba (2016)	Deferasirox	Oral	20–40 mg/kg/day	Once a day	Gastrointestinal disturbances and skin rash	[79]
Piga et al. (2006)	Deferasirox	Oral	10 or 20 mg/kg/day	Once a day	—	[75]
Porter et al. (2008)	Deferasirox	Oral	5, 10, 20, and 30 mg/kg/day based on liver iron concentration	Once a day	Skin rash, increased serum creatinine, elevated alanine aminotransferase, auditory disorders, and gastrointestinal events including abdominal pain, nausea, vomiting, diarrhea, and constipation	[73]
Taher et al. (2009)	Deferasirox	Oral	10 or 20 mg/kg/day	Once a day	Vomiting, skin rash, nausea, increased alanine aminotransferase, increased serum creatinine, and obstructive jaundice	[67]
Taher et al. (2017)	Deferasirox	Oral	14 or 20 mg/kg/day	Once a day	Diarrhea, renal events, increased urine protein/creatinine ratio, abdominal pain, vomiting, and nausea	[81]
Vichinsky et al. (2007)	Deferasirox	Oral	10–30 mg/kg	Once a day	Abdominal pain, nausea, vomiting, diarrhea, back pain, skin rash, headache, upper respiratory tract infection, gastrointestinal disorders, sickle cell disease with crisis, increases in serum creatinine, and elevated alanine aminotransferase	[72]

N/A, not available; TDT, transfusion-dependent *β*-thalassemia.

**Table 4:** Summary of the findings on combination therapy.

Author (year)	Property	Route	Dose	Schedule	Side effects	Reference
Voskaridou et al. (2012)	Deferoxamine and deferasirox	DFO: subcutaneous DFX: oral	DFO: 2500 mg/day DFX: 30 mg/kg/day	DFO: 4 days every week DFX: 7 days per week	Well-tolerated combination regimen and no adverse events	[134]
Lal et al. (2013)	Deferoxamine and deferasirox	DFO: subcutaneous DFX: oral	DFO: 35–50 mg/kg DFX: 20–30 mg/kg	DFO: 3–7 days/week DFX: daily	Recurrent abdominal pain, infection of an implanted port with *Trichosporon asahii* ascites, diarrhea, and leukocytosis	[131]
Cassinerio et al. (2014)	Deferoxamine and deferasirox	DFO: infusion therapy DFX: oral	DFO: 32 ± 4 mg/kg/day DFX: 20 ± 2 mg/kg/day (based on ferritin level and patient tolerability, doses were gradually increased)	DFO: 3–4 days a week DFX: daily	Data available at 1 year showed no alteration of renal/hepatic function and no adverse events.	[130]
Takpradit et al. (2021)	Deferoxamine and deferasirox	DFO: continuous subcutaneous or intravenous DFX: oral	DFO: 18–40 mg/kg/day DFX: 20–40 mg/kg/day	DFO: 3–6 days/week DFX: twice a day	No treatment-related complications	[132]
Eghbali et al. (2019)	Deferoxamine and deferasirox	DFO: subcutaneous DFX: oral	DFO: 50 mg/kg DFX: 30 mg/kg	DFO: 5 days a week DFX: daily	Mild gastrointestinal symptoms — Levels of total bilirubin were significantly higher in patients who received the combination therapy. — No patient died. No patient was withdrawn due to severe adverse effects.	[133]
Alymara et al. (2004)	Deferoxamine and deferiprone	DFO: subcutaneous DFP: oral	DFO: 40–50 mg/kg/day DFP: 60 mg/kg/day	DFO: 8–12 constant hours, 4–6 days a week DFP: three divided doses, 6 days a week	Gastrointestinal disorders, including nausea and vomiting, anorexia, weight loss, elevations in aspartate aminotransferase and alanine aminotransferase serum levels, neutropenia, stiffness of the elbow, pain of the knee joints, taste disorders, dizziness, and fatigue	[138]
Chuang et al. (2020)	Deferoxamine and deferiprone	DFO: intravenous DFP: oral	DFO: 50 mg/kg/day DFP: 100 mg/kg/day	DFO: continuous 24-hour infusion DFP: three divided doses, both 7 days a week	Elevation of serum alanine transaminase and elevation of serum creatinine	[137]
Porcu et al. (2007)	Deferoxamine and deferiprone	DFO: subcutaneous DFP: oral	DFO: 500 mg/day DFP: 75 mg/kg/day	DFO: N/A DFP: three divided doses	No relevant side effects related to deferiprone have been reported.	[40]
Songdej et al. (2015)	Deferoxamine and deferiprone	DFO: subcutaneous DFP: oral	DFO: 40 mg/kg/dose DFP: 50–100 mg/kg/day	DFO: 8–12 hours twice weekly DFP: 3–4 once a day	Elevated alanine transaminase, neutropenia, thrombocytopenia, cholecystitis with infection-associated hemophagocytic syndrome, proteinuria, elevated serum creatinine, and gastrointestinal discomfort	[135]
Tauchenová et al. (2016)	Deferoxamine and deferiprone	DFO: intravenous DFP: oral	DFO: 35 mg/kg/day DFP: 75 mg/kg/day	DFO: 12 divided doses DFP: three divided doses, both daily	Leukopenia	[136]
DivakarJose et al. (2021)	Deferoxamine and deferiprone	DFP: oral DFX: not mentioned	DFO: 30 mg/kg/day DFP: 75 mg/kg/day	DFO: three divided doses DFP: three divided doses, both daily	—	[139]
Pathare et al. (2004)	Deferoxamine and deferiprone	DFX: subcutaneous DFP: oral	DFX: 40 mg/kg/day DFP: 75 mg/kg/day	8–10 hours on 4–5 nights weekly/three divided doses	Severe upset gastrointestinal tract, raised liver enzymes, death (sepsis), needing bone marrow transplantation, and agranulocytosis	[143]
Elalfy et al. (2015)	Deferasirox and deferiprone	DFX: oral DFP: oral	DFX: 30–40 mg/kg/day DFP: 75–100 mg/kg/day	DFX: once a day DFP: divided into two doses	Gastrointestinal manifestations, such as nausea and mild abdominal pain, and neutropenia (mild–moderate)	[152]
Parakh et al. (2017)	Deferasirox and deferiprone	DFX: oral DFP: oral	DFX: 5–40 mg/kg DFP: 10–100 mg/kg/day	DFX: once a day DFP: three doses	Arthropathy, deranged creatinine level (moderate increase), gastrointestinal adverse events, including abdominal pain, nausea, and vomiting, and joint symptoms, including arthralgia involving the knee joint	[147]
Lin et al. (2019)	Deferasirox and deferiprone	DFX: oral DFP: oral	DFX: 30 mg/kg DFP: 40 mg/kg	DFX: once a day DFP: two doses	Significant increase in fecal iron excretion increases the risk of renal toxicity	[151]
Karami et al. (2017)	Deferasirox and deferiprone	DFX: oral DFP: oral	DFX: 30 mg/kg/day DFP: 75 mg/kg/day	DFX: once a day DFP: three doses	Neutropenia and diarrhea	[149]

N/A, not available.
